# Letter contrast sensitivity validation

**DOI:** 10.1111/opo.13555

**Published:** 2025-08-16

**Authors:** Michel Guillon, Pasquale Pepe, Jessie Hull, Rajaraman Suryakumar

**Affiliations:** ^1^ Ocular Technology Group – International London UK; ^2^ Alcon Research LLC Fort Worth Texas USA

**Keywords:** computerised letter test, contrast sensitivity, sinusoidal test, test–retest repeatability

## Abstract

**Purpose:**

Contrast sensitivity (CS) is an important tool for evaluating functional vision. The Ocular Technology Group—International Vision Suite computerised letter CS test can evaluate multiple spatial frequencies, assessing the entire CS function. This clinical study validated the letter and sinusoidal CS methods by determining their repeatability using multiple spatial frequency settings and different lighting conditions.

**Methods:**

The single‐arm, prospective study compared the repeatability of CS measurements at two separate visits using three methods: computerised letter CS, computerised sinusoidal CS (M&S Technologies) and printed sinusoidal CS (VectorVision). Measurements were performed under photopic (without glare) or mesopic (with or without glare) conditions. Repeatability was assessed in 20 participants by comparing the mean and 95% CI of the difference between the measurements.

**Results:**

Test variance was significantly lower for letter CS compared with the M&S or VectorVision instruments at most spatial frequencies. Letter CS measurements had a substantially lower precision‐to‐tolerance ratio compared with the M&S or VectorVision and required the smallest estimated sample size to achieve a precision‐to‐tolerance ratio of 0.25.

**Conclusions:**

Overall, the computerised letter method for assessing CS function was highly repeatable and had significantly lower test variance when compared with either of the sinusoidal instruments.

**Key points**

- The computerised letter method for assessing the contrast sensitivity function was highly repeatable and had significantly less variability compared with clinical sinusoidal grating techniques at most spatial frequencies and lighting conditions.
- Good repeatability makes the computerised letter method a good option for clinical studies because the lower test variability requires smaller sample sizes to achieve the same statistical power as investigations using sinusoidal methods.
- The current study demonstrated that a substantially smaller study population size would be needed to achieve a desired precision‐to‐tolerance ratio when using the letter contrast sensitivity method compared with clinical sinusoidal techniques.

## INTRODUCTION

Contrast sensitivity (CS) provides information about functional vision and is an important assessment of visual performance. CS assessment may help detect and evaluate the progression of ocular diseases, such as glaucoma and cataract,^1^, ^2^, ^3^ and may be used as a tool for optimising treatment, refractive surgery and devices.^4^ Furthermore, CS assessments may be particularly important in older patients because of a decline in CS associated with age.^5^, ^6^

Standard visual acuity tests measure resolution and provide an assessment of the smallest size black‐on‐white letter that can be read. However, individuals encounter everyday objects of varying sizes and contrasts (the luminance of an object compared with the background) that cannot be measured adequately using visual acuity tests. Two popular methods used to measure CS are sinusoidal targets and letter optotypes. Sinusoidal wave gratings of different sizes (spatial frequencies) and contrast can be used to determine the patient's CS function. The ability to detect lower contrast for any grating represents higher CS.^4^ Spatial frequencies are measured in cycles per degree of visual angle (cpd).

Sinusoidal assessments and letter methods have fundamental differences and may not provide equivalent information about CS.^7^ Typically, sinusoidal grating charts measure CS at multiple spatial frequencies. Letter tests, such as the Pelli‐Robson chart, can measure CS using letters of constant size and varying degrees of contrast.^8^ Additionally, other clinical CS assessments that could be self‐administered and provide similar test–retest repeatability when compared with a Pelli‐Robson chart (e.g., SpotChecks, Precision Vision and precision‐vision.com) have been developed.^9^ The stability of CS for relatively large objects is discussed further by Vu et al.^9^

Both letter and sinusoidal CS tests are available in computerised and printed form. Computerised tests can be time‐consuming and require training. Although printed sinusoidal CS assessment is a quick clinical test, the limited presentations available at each spatial frequency narrow its resolution. Additionally, printed charts are often displayed on a backlit light box, potentially reducing the lowest threshold of contrast that could be displayed. Modern computerised CS tests have circumvented this issue by using liquid crystal displays (LCD) or light emitting diode (LED) displays that have a large dynamic range of contrast representation.

In clinical settings, there is a need for CS measurements to be fast, repeatable and valid. Contrast letter tests, such as the printed Pelli‐Robson test, are easy to use because of patients' familiarity with reading letters.^8^ Letter tests have been shown to be highly reproducible; several studies have reported similar repeatability when assessing CS using the Pelli‐Robson chart.^10^, ^11^, ^12^, ^13^, ^14^ Little learning effect is associated with letter CS because of the familiarity of the task; hence, the method is directly applicable to clinical trials involving a significant number of participants. Furthermore, previous studies have demonstrated that the computer‐generated letter‐based M&S CS test (manufacturer details provided in the method section) was comparable with the Pelli‐Robson chart, with a mean difference between the two testing methods of 0.03 log units.^15^ A computerised letter‐based method using the Ocular Technology Group–International (OTG‐I) Vision Suite can evaluate multiple spatial frequencies, as well as contrast, thereby measuring the entire CS function.

The purpose of this study was to validate letter and sinusoidal CS methods by determining their repeatability in a representative presbyopic population using multiple spatial frequency settings and different lighting conditions. Repeatability of the computerised letter method was compared with two commercially available sinusoidal pattern methods (M&S Technologies and VectorVision).

## METHODS

### Study design

This was a single‐arm, prospective, controlled, crossover, repeated measure study. Participants completed three visits during the study period (January to February 2021). Visit 1 included screening and enrolment. Visits 2 and 3 included test measurements. During visits 2 and 3, the order of using the three measurement methods was the same for each participant to keep testing conditions identical. Measurements were taken from the dominant eye using the best sphero‐cylindrical spectacle correction determined at the study onset. The dominant eye was determined using the pointing method or the sensitivity‐to‐blur method. The nondominant eye was occluded with a frosted lens to maintain similar luminance in both eyes at the pupil level. Eligible participants had corrected distance visual acuity equal to or better than 6/6 in each eye. Other inclusion criteria were 40–50 years of age, sphere between *−*6.00 and +4.00 D, astigmatism ≤3.00 D and near addition +0.50 to +2.50 D. Excluded from the study were participants with conditions that would contraindicate contact lens wear, monocular participants and any moderate or severe ocular condition observed during the slit‐lamp examination at the enrolment visit.

This study was performed in compliance with the ethical principles of the Declaration of Helsinki and Good Clinical Practice, the International Organisation for Standardisation ISO14155:2015 and the applicable US Food and Drug Administration FDA21 Code of Federal Regulations.

### Assessments

**FIGURE 1:**
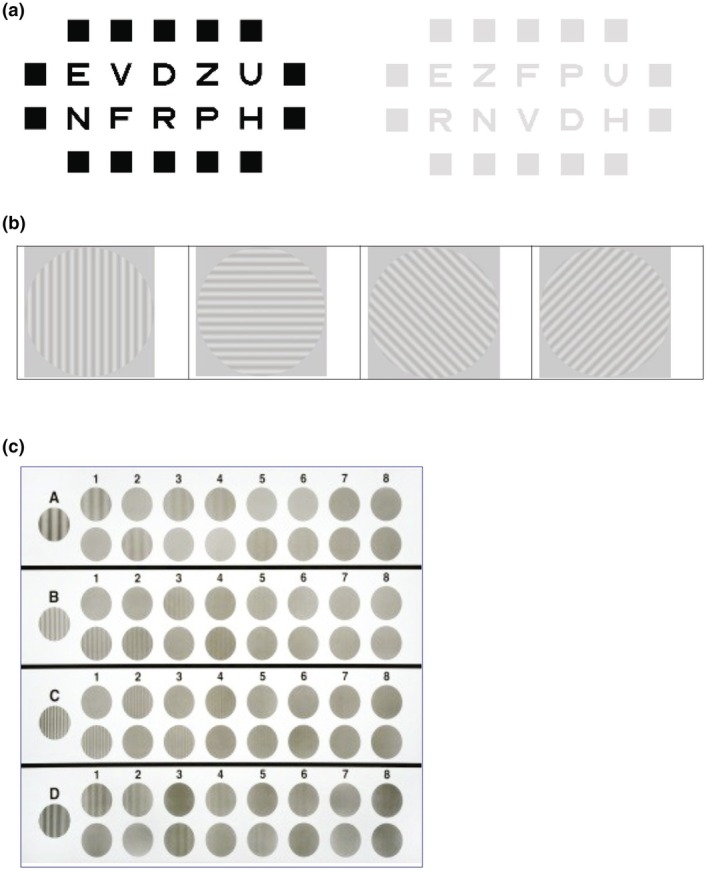
Contrast sensitivity measurements using computerised letter method (a), M&S Technologies method (b) and VectorVision test method (c).

CS was measured in the dominant eye under photopic conditions (85 cd/m^2^) without glare and under mesopic conditions (3 cd/m^2^) both with and without glare. Assessments were performed using three methods: (i) computerised letter CS using the Vision Suite (OTG‐I; otgi.co/) letter contrast test at 4 m (1.5, 3, 6, 12 and 18 cpd; Figure 1a), (ii) computerised sinusoidal CS using the Clinical Trial Suite (M&S Technologies; mstech‐eyes.com) linear sine wave grating test at 2.5 m (1.5, 3, 6, 12 and 18 cpd; Figure 1b) and (iii) a printed sinusoidal CVS‐1000 (VectorVision; vectorvision.com/) standardised CS test at 2.5 m (3, 6, 12 and 18 cpd; Figure 1c). Standard commercial instrument glare sources were used for the M&S and VectorVision tests. For OTG‐I glare sources, four spotlights located at each corner of the screen were used to mimic the M&S setup. The manufacturers' measurement procedures were followed for all three methods; standard procedures per M&S Technologies and VectorVision instruction manuals were used for the sinusoidal tests. For the M&S sinusoidal test and for each spatial frequency, participants were shown targets and instructed to indicate which direction the target was pointing; testing started with the highest contrast and ended with the lowest contrast level. The VectorVision sinusoidal test was a psychophysical evaluation using the 2‐alternative forced‐choice, descending limits method. The patch diameter was 14 cm for the M&S test and 3.8 cm for the VectorVision test. The contrast range was 100% to 0.8% and 90% to 0.5% for the M&S and VectorVision tests, respectively.

For the computerised letter test, for each contrast level, 10 computerised letters per the International Organization for Standardization were presented in two rows with crowding blocks to maintain a constant difficulty throughout the chart (Figure 1a). Spatial frequency was allocated per the standard approach for letter contrast sensitivity described by McAnany and Alexander.^7^ Briefly, VA letters have a ratio of 5:1 for the height versus thickness of the letter element and 4:1 for the letter width versus thickness (with the exception of square Sloan letters, for which the ratio for horizontal and the thickness of the element is 5:1). The spatial frequency was calculated by taking one cycle to correspond with the thickness of the letter element plus an equal surround value, following the National Academy of Sciences 1980 guidelines. The contrast threshold for the letter CS test was calculated using the Weber method,^17^ and the contrast progression was logarithmic with each step equal to 0.1 log contrast. The log contrast ranged from 2.0 to −0.4. The threshold was determined to the nearest letter and participants were asked to read the letters until at least six of the 10 letters were not read correctly. The percentage threshold for the M&S and VectorVision tests was calculated using the Michelson method.^17^ All data were converted to the log of decimal contrast units.

### Statistical analysis

Repeatability was assessed by comparing the mean and 95% CI of the difference between the measurements at visits 2 and 3 and calculating the variance between the measurements. The difference between visits 2 and 3 was calculated for each participant separately (within participant). The variance for the test was obtained by calculating the square of the observed standard deviation. Repeatability for each method was evaluated using a linear mixed model or a generalised linear mixed model. Contrast sensitivity in log scale (normally distributed at each spatial frequency) was analysed using a linear mixed model. The percentage scale, which was not normally distributed, was analysed by means of the best fitting model, which was a gamma distributed mixed model with log link according to the right skewed distributions of the data at each spatial frequency. Noninferiority of the letter test compared with the two control methods was performed using the paired Pitman–Morgan ratio of variances test.

The precision‐to‐tolerance (P/T) ratio was calculated with a margin of clinical equivalence of ±0.2 log units using the equation: P/T = (6σ_e_)/(USL − LSL), where USL is the upper specification limit (upper limit of clinical significance) and LSL is the lower specification limit. The measurement standard deviation was multiplied by 6 to give the ‘spread’ of the measurement error. This spread is divided by the tolerance (USL – LSL) to obtain the P/T value. The P/T value can be interpreted as the spread of the tolerance that is ‘consumed’ by the measurement error. Calculation of the sample size to achieve a desired population P/T ratio was as follows:

$$\sqrt{n}=\frac{P/T_{\text{patient}}}{P/T_{\text{population}}}$$

Repeatability of the letter CS, M&S and VectorVision tests was also evaluated using the Bland–Altman plot analysis (Figures S1–S3). The difference between two pairs of measurements was plotted against the mean of the two measurements, and 95% of the data points were expected to lie within ±2 standard deviations of the mean difference (i.e., the upper and lower limits of agreement).

## RESULTS

### Participants

The study included 20 participants with a mean ± SD age of 43.6 ± 2.8 years (range, 40–49 years). The majority were female (65%). Most participants had myopia (mean ± SD sphere, −1.53 ± 2.44 D in the dominant eye) with low levels of astigmatism (mean ± SD cylinder −0.59 ± 0.42 D in the dominant eye). For the dominant eye, mean ± SD distance and near Snellen decimal equivalent visual acuity were 1.14 ± 0.13 and 0.82 ± 0.09, respectively.

### Contrast sensitivity repeatability

**FIGURE 2:**
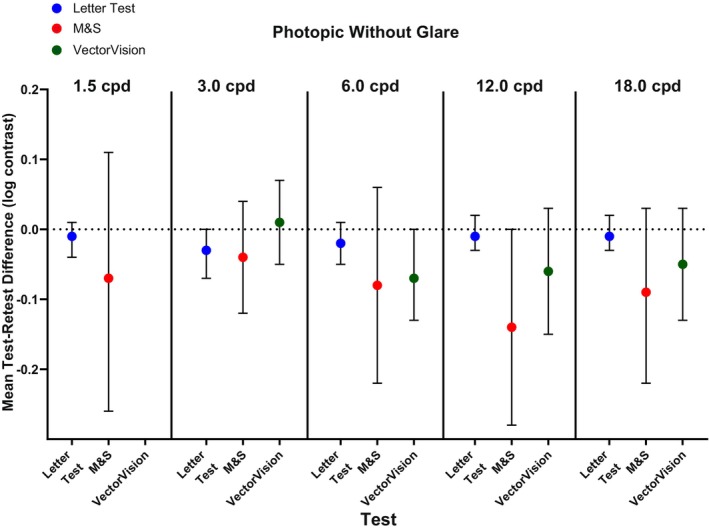
Mean test–retest difference representing contrast sensitivity threshold repeatability under photopic conditions without glare for letter test, M&S Technologies and VectorVision test methods. Error bars represent 95% confidence intervals. cpd, cycles per degree of visual angle.

The CS threshold values under photopic conditions without glare and under mesopic conditions with or without glare at the two separate visits are summarised in Tables S1–S3. Under photopic conditions, test variance was significantly lower for letter CS compared with the M&S test method (*p* ≤ 0.001) and for letter CS compared with the VectorVision test method (*p* ≤ 0.02; Table 1) at all spatial frequencies. Mean test–retest differences were −0.03 to −0.01 (95% CI amplitudes for the different spatial frequencies ranged from 0.05 to 0.07) log contrast for the letter CS test method, −0.14 to −0.04 (95% CI amplitudes for the different spatial frequencies ranged from 0.16 to 0.37) log contrast for the M&S test method and − 0.07 to +0.01 (95% CI amplitudes for the different spatial frequencies ranged from 0.12 to 0.18) log contrast for the VectorVision test method (Figure 2).

**TABLE 1:** Contrast sensitivity threshold repeatability and variance ratio under photopic conditions without glare.

Test method	1.5 cpd	3.0 cpd	6.0 cpd	12.0 cpd	18.0 cpd
**Computerised letter test**					
Mean test–retest difference ± SD, log contrast	−0.01 ± 0.06	−0.03 ± 0.08	−0.02 ± 0.07	−0.01 ± 0.07	−0.01 ± 0.06
**M&S Technologies sinusoidal test**					
Mean test–retest difference ± SD, log contrast	−0.07 ± 0.41	−0.04 ± 0.17	−0.08 ± 0.31	−0.14 ± 0.31	−0.09 ± 0.28
Letter/Sinusoidal variance ratio	0.024	0.198	0.051	0.044	0.048
*p* value	<0.001	0.001	<0.001	<0.001	<0.001
**VectorVision sinusoidal test**					
Mean test–retest difference ± SD, log contrast		0.01 ± 0.13	−0.07 ± 0.14	−0.06 ± 0.21	−0.05 ± 0.18
Letter/Sinusoidal variance ratio		0.334	0.263	0.098	0.118
*p* value		0.02	0.004	<0.001	0.001

Abbreviation: cpd, cycles per degree of visual angle.

**FIGURE 3:**
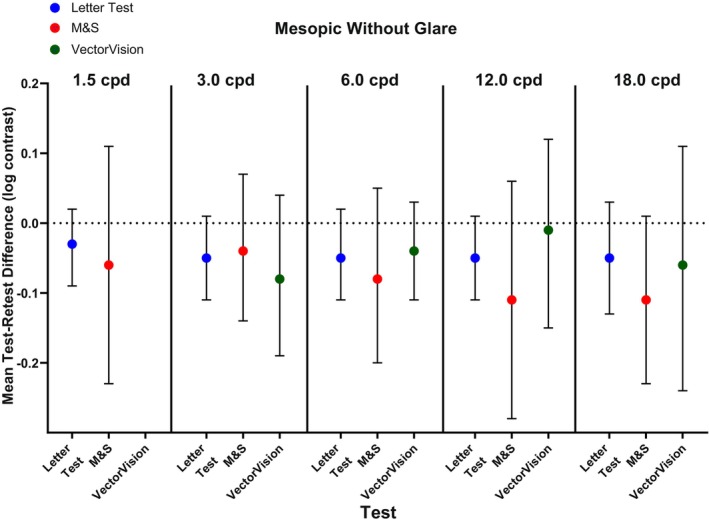
Mean test–retest difference representing contrast sensitivity threshold repeatability under mesopic conditions without glare for letter test, M&S Technologies and VectorVision test methods. Error bars represent 95% confidence intervals. cpd, cycles per degree of visual angle.

Under mesopic conditions without glare, test variance was significantly lower for letter CS compared with the M&S test method at most spatial frequencies (*p* ≤ 0.01) except for 18 cpd (*p* = 0.07) and for letter CS compared with the VectorVision test method for most spatial frequencies (*p* ≤ 0.004) except for 6 cpd (*p* = 0.97; Table 2). Mean test–retest differences were −0.05 to −0.03 (95% CI amplitudes for the different spatial frequencies ranged from 0.11 to 0.16) log contrast for the letter CS test method, −0.11 to −0.04 (95% CI amplitudes for the different spatial frequencies ranged from 0.21 to 0.34) log contrast for the M&S test method, and − 0.08 to −0.01 (95% CI amplitudes for the different spatial frequencies ranged from 0.14 to 0.35) log contrast for the VectorVision test method (Figure 3).

**TABLE 2:** Contrast sensitivity threshold repeatability and variance ratio under mesopic conditions without glare.

Test method	1.5 cpd	3.0 cpd	6.0 cpd	12.0 cpd	18.0 cpd
**Computerised letter test**					
Mean test–retest difference ± SD, log contrast	−0.03 ± 0.13	−0.05 ± 0.13	−0.05 ± 0.15	−0.05 ± 0.14	−0.05 ± 0.18
**M&S Technologies sinusoidal test**					
Mean test–retest difference ± SD, log contrast	−0.06 ± 0.38	−0.04 ± 0.24	−0.08 ± 0.28	−0.11 ± 0.38	−0.11 ± 0.27
Letter/Sinusoidal variance ratio	0.110	0.293	0.281	0.141	0.428
*p* value	<0.001	0.01	0.006	<0.001	0.07
**VectorVision sinusoidal test**					
Mean test–retest difference ± SD, log contrast		−0.08 ± 0.24	−0.04 ± 0.15	−0.01 ± 0.30	−0.06 ± 0.39
Letter/Sinusoidal variance ratio		0.276	1.016	0.219	0.201
*p* value		0.004	0.97	0.001	0.001

Abbreviation: cpd, cycles per degree of visual angle.

**FIGURE 4:**
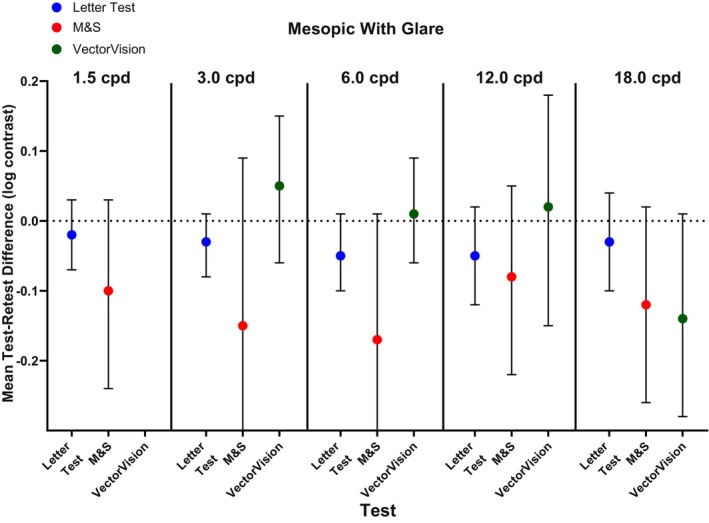
Mean test–retest difference representing contrast sensitivity threshold repeatability under mesopic conditions with glare for letter test, M&S Technologies and VectorVision test methods. Error bars represent 95% confidence intervals. cpd, cycles per degree of visual angle.

Under mesopic conditions with glare, test variance was significantly lower for letter CS compared with M&S test measurements at all spatial frequencies (*p* ≤ 0.007) and for letter CS compared with the VectorVision test method at most spatial frequencies (*p* ≤ 0.004) except 6 cpd (*p* = 0.16; Table 3). Mean test–retest differences were −0.05 to −0.02 (95% CI, 0.09 to 0.14) log contrast for the letter CS test method, −0.17 to −0.08 (95% CI, 0.27 to 0.48) log contrast for the M&S test method and − 0.14 to 0.05 (95% CI, 0.15 to 0.33) log contrast for the VectorVision test method (Figure 4).

**TABLE 3:** Contrast sensitivity threshold repeatability and variance ratio under mesopic conditions with glare.

Test method	1.5 cpd	3.0 cpd	6.0 cpd	12.0 cpd	18.0 cpd
**Computerised letter test**					
Mean test–retest difference ± SD, log contrast	−0.02 ± 0.12	−0.03 ± 0.10	−0.05 ± 0.12	−0.05 ± 0.16	−0.03 ± 0.16
**M&S Technologies sinusoidal test**					
Mean test–retest difference ± SD, log contrast	−0.10 ± 0.29	−0.15 ± 0.53	−0.17 ± 0.39	−0.08 ± 0.30	−0.12 ± 0.30
Letter/Sinusoidal variance ratio	0.156	0.037	0.089	0.268	0.278
*p* value	<0.001	0.001	<0.001	0.006	0.007
**VectorVision sinusoidal test**					
Mean test–retest difference ± SD, log contrast		0.05 ± 0.23	0.01 ± 0.16	0.02 ± 0.36	−0.14 ± 0.32
Letter/Sinusoidal variance ratio		0.189	0.517	0.193	0.254
*p* value		0.001	0.16	0.001	0.004

Abbreviation: cpd, cycles per degree of visual angle.

The letter CS measurements had a substantially lower P/T ratio compared with the M&S and VectorVision test methods at all spatial frequencies, confirming its greater repeatability for all testing conditions (Table 4). To achieve a P/T ratio of 0.25, the estimated sample size was smallest for the letter CS method (≤20 participants under photopic conditions; ≤111 participants under mesopic conditions without glare and ≤ 92 participants under mesopic conditions with glare) compared with the M&S method (≤599 participants under photopic conditions; ≤524 participants under mesopic conditions without glare and ≤ 1007 participants under mesopic conditions with glare) or the VectorVision method (≤156 participants under photopic conditions; ≤552 participants under mesopic conditions without glare and ≤ 466 participants under mesopic conditions with glare).

**TABLE 4:** Contrast sensitivity precision‐to‐tolerance ratio.

Test conditions	Method	1.5 cpd	3.0 cpd	6.0 cpd	12.0 cpd	18.0 cpd
Photopic without glare	Letter test	0.95	1.13	1.06	0.98	0.93
	M&S	6.12	2.53	4.70	4.65	4.24
	VectorVision		1.95	2.07	3.13	2.69
Mesopic without glare	Letter test	1.91	1.92	2.26	2.11	2.63
	M&S	5.72	3.53	4.25	5.63	4.02
	VectorVision		3.65	2.24	4.51	5.87
Mesopic with glare	Letter test	1.74	1.53	1.75	2.37	2.40
	M&S	4.42	7.93	5.87	4.56	4.55
	VectorVision		3.52	2.44	5.39	4.77

Abbreviation: cpd, cycles per degree of visual angle.

## DISCUSSION

Contrast sensitivity, or the ability to detect differences in relative brightness between objects and their background, is an important characteristic of visual function. CS declines with age and may be an indicator of ocular disease,^3^, ^4^, ^5^, ^6^ making it an important factor to consider when evaluating patients' visual function. In this study, a computerised letter method for assessing CS function was highly repeatable and had significantly lower test variance compared with commercially available sinusoidal pattern methods at most spatial frequencies.

For the computerised letter CS test, the mean test–retest differences were − 0.03 to −0.01, −0.05 to −0.03 and − 0.05 to −0.02 log contrast for photopic, mesopic without glare and mesopic with glare conditions, respectively. For the M&S sinusoidal test, the mean test–retest differences were − 0.14 to −0.04, −0.11 to −0.04 and − 0.17 to −0.08 log contrast for photopic, mesopic without glare and mesopic with glare conditions, respectively. For the VectorVision sinusoidal test, the mean test–retest differences were − 0.07 to 0.01, −0.08 to −0.01 and − 0.14 to 0.05 log contrast for photopic, mesopic without glare and mesopic with glare conditions, respectively. These results were consistent with an average test–retest difference of −0.07 and − 0.014 log CS reported with the sinusoidal VectorVision 1000E chart for children and adults, respectively.^18^ A test–retest mean difference of 0.02 log contrast was noted for the traditional letter CS methods (Mars and Pelli‐Robson tests) and no significant differences were indicated for test–retest repeatability using the M&S method.^11^, ^19^

Based on the analysis of the results in this study, repeatability was significantly better for the letter CS method compared with the sinusoidal M&S method at most spatial frequencies and lighting conditions (*p* < 0.05). The only nonsignificant difference was reported at an 18 cpd spatial frequency under mesopic conditions without glare (*p* = 0.07). Furthermore, the letter CS method had significantly better repeatability compared with the sinusoidal VectorVision method at all spatial frequencies under photopic conditions (*p* < 0.05) and at most spatial frequencies under mesopic conditions with or without glare (*p* < 0.05 for 3, 12 and 18 cpd). These findings are consistent with previous reports that demonstrated greater reproducibility for letter‐based tests compared with sinusoidal gratings charts.^14^, ^20^

Although CS has traditionally been assessed using sinusoidal gratings methods,^4^ the letter‐based CS assessment is the preferred method for clinical settings. Letter CS measurements are easy to perform for a practitioner or technician, with minimal training required. Furthermore, letter recognition tests may be easier for patients compared with detecting sinusoidal pattern differences because reading letters is a familiar task while detecting sinusoidal contrast difference is not. To provide familiarity with sinusoidal patterns, training sessions would need to be added to the clinical trial protocol, requiring additional time and cost.

Good repeatability also makes the letter CS method suitable for clinical studies because the lower variance demonstrated with this method may require smaller sample sizes to achieve the same statistical power as studies using sinusoidal CS. Measurement repeatability is critical when carrying out a clinical trial comparing a new device or treatment with a control condition. When testing clinical noninferiority or superiority of a new test device, the sample size required for demonstrating statistical power is calculated using the margin of clinical equivalence and test repeatability.^21^ The better the test repeatability, the smaller the sample size needed to detect the difference between devices or treatments for a fixed margin of equivalence. The current study demonstrated that a substantially smaller population size would be needed to achieve a desired P/T ratio when using the letter CS method versus the M&S or VectorVision instruments. The results of this study make it possible to calculate the required sample size for any future clinical study involving letter contrast sensitivity.

Previous findings suggested that the Pelli‐Robson chart and Cambridge gratings were a good measure of CS at low and intermediate spatial frequencies, whereas other types of tests (e.g., Regan low contrast chart and Small Letter Contrast Test) were optimal at medium to high spatial frequencies.^20^, ^22^ In the current study, multiple spatial frequencies were used for CS assessment. Measuring CS at a wide range of spatial frequencies can detect subtle changes in vision and help identify impairments and ocular conditions that selectively affect CS at different spatial frequencies. For example, amblyopia can reduce sensitivity at high spatial frequencies, whereas glare can reduce sensitivity at low spatial frequencies.

Strengths of this study included testing both letter and sinusoidal methods under multiple spatial frequency settings and lighting conditions. Both with‐glare and without‐glare conditions were tested for mesopic CS. Limitations of this study included a relatively small sample size and limited age range of the participants. Although the time to carry out the tests was not recorded, the overall experience was that the letter CS test following the testing instructions was faster than the M&S sinusoidal test because of the lack of familiarity with the sinusoidal patterns. The VectorVision test was the fastest test to perform because of the very limited options at each spatial frequency; however, contrast resolution using this instrument was low. Future studies evaluating CS assessment methods should include a larger and more diverse population as well as patients in different age groups. Additionally, future studies can assess the correlations between letter CS at specific spatial frequencies versus letter CS measured using sinusoidal gratings.

In conclusion, the results of this study demonstrated significantly better repeatability when measuring CS using the computerised letter test method compared with sinusoidal grating methods for most testing conditions.

## AUTHOR CONTRIBUTIONS

**Michel Guillon:** Conceptualization (equal); data curation (equal); funding acquisition (equal); investigation (equal); methodology (equal); project administration (equal); supervision (equal); writing – review and editing (equal). **Pasquale Pepe:** Data curation (equal); formal analysis (equal); methodology (equal); validation (equal); writing – review and editing (equal). **Jessie Hull:** Conceptualization (equal); writing – review and editing (equal). **Rajaraman Suryakumar:** Conceptualization (equal); data curation (equal); funding acquisition (equal); methodology (equal); writing – review and editing (equal).

## FUNDING INFORMATION

This study was funded by Alcon Research LLC.

## CONFLICT OF INTEREST STATEMENT

Michel Guillon: Owner of Optometric Technology Group Ltd., which developed Ocular Technology Group—International Vision Suite, and received funding from Alcon Research LLC to carry out the study. Pasquale Pepe: Received funding from Alcon Research LLC to carry out this study. Jessie Hull and Rajaraman Suryakumar are employees of Alcon Research LLC.

## PATIENT CONSENT STATEMENT

All enrolled participants signed the consent form.

## CLINICAL TRIAL REGISTRATION

The trial was registered on the ISCRTN public website as per ethics committee condition of approval.

## Supporting information


Data S1:
