# Supplementary material for: Letter contrast sensitivity validation
Source: Ophthalmic Physiol Opt. 2025 Aug 16;45(6):1317–25. doi: 10.1111/opo.13555 (PMC12357228; doi:10.1111/opo.13555)
Supplement: Supplementary file 1 — Data S1: [file OPO-45-1317-s001.zip › opo13555-sup-0001-Supinfo.docx]

**Supplemental Table 1.** Mean CS threshold under photopic conditions without glare. cpd, cycles per degree of visual angle.

| **Mean CS threshold ± SD, log contrast** | **1.5 cpd** | **3.0 cpd** | **6.0 cpd** | **12.0 cpd** | **18.0 cpd** |
| --- | --- | --- | --- | --- | --- |
| **Computerised letter test** |  |  |  |  |  |
| Visit 1 | −1.88±0.07 | −1.87±0.07 | −1.74±0.12 | −1.51±0.11 | −1.35±0.12 |
| Visit 2 | −1.90±0.08 | −1.91±0.09 | −1.75±0.12 | −1.52±0.11 | −1.36±0.11 |
| **M&S Technologies sinusoidal test** |  |  |  |  |  |
| Visit 1 | −1.94±0.31 | −2.24±0.17 | −2.03±0.24 | −1.58±0.39 | −1.15±0.35 |
| Visit 2 | −2.02±0.44 | −2.27±0.15 | −2.11±0.35 | −1.71±0.24 | −1.25±0.28 |
| **VectorVision sinusoidal test** |  |  |  |  |  |
| Visit 1 |  | −1.89±0.16 | −2.05±0.15 | −1.68±0.21 | −1.26±0.23 |
| Visit 2 |  | −1.89±0.12 | −2.12±0.09 | −1.74±0.15 | −1.31±0.13 |

**Supplemental Table 2.** Mean CS threshold under mesopic conditions with glare. cpd, cycles per degree of visual angle.

| **Mean CS threshold ± SD, log contrast** | **1.5 cpd** | **3.0 cpd** | **6.0 cpd** | **12.0 cpd** | **18.0 cpd** |
| --- | --- | --- | --- | --- | --- |
| **Computerised letter test** |  |  |  |  |  |
| Visit 1 | −1.52±0.15 | −1.45±0.17 | −1.23±0.17 | −0.87±0.20 | −0.63±0.19 |
| Visit 2 | −1.54±0.16 | −1.49±0.12 | −1.27±0.13 | −0.92±0.19 | −0.66±0.16 |
| **M&S Technologies sinusoidal test** |  |  |  |  |  |
| Visit 1 | −1.21±0.25 | −1.54±0.45 | −1.26±0.36 | −0.72±0.34 | −0.32±0.27 |
| Visit 2 | −1.31±0.29 | −1.69±0.42 | −1.43±0.39 | −0.80±0.33 | −0.44±0.32 |
| **VectorVision sinusoidal test** |  |  |  |  |  |
| Visit 1 |  | −1.61±0.20 | −1.60±0.22 | −0.96±0.39 | −0.40±0.34 |
| Visit 2 |  | −1.56±0.18 | −1.59±0.19 | −0.94±0.41 | −0.53±0.40 |

**Supplemental Table 3.** Mean CS threshold under mesopic conditions without glare. cpd, cycles per degree of visual angle.

| **Mean CS threshold ± SD, log contrast** | **1.5 cpd** | **3.0 cpd** | **6.0 cpd** | **12.0 cpd** | **18.0 cpd** |
| --- | --- | --- | --- | --- | --- |
| **Computerised letter test** |  |  |  |  |  |
| Visit 1 | −1.53±0.15 | −1.47±0.16 | −1.24±0.17 | −0.89±0.19 | −0.65±0.20 |
| Visit 2 | −1.57±0.12 | −1.52±0.12 | −1.29±0.12 | −0.94±0.15 | −0.69±0.14 |
| **M&S Technologies sinusoidal test** |  |  |  |  |  |
| Visit 1 | −1.84±0.40 | −2.08±0.26 | −1.64±0.31 | −0.92±0.38 | −0.54±0.22 |
| Visit 2 | −1.90±0.36 | −2.12±0.24 | −1.71±0.28 | −1.03±0.35 | −0.64±0.22 |
| **VectorVision sinusoidal test** |  |  |  |  |  |
| Visit 1 |  | −1.52±0.20 | −1.60±0.22 | −1.08±0.31 | −0.44±0.31 |
| Visit 2 |  | −1.59±0.18 | −1.64±0.20 | −1.10±0.35 | −0.50±0.38 |
