# Supplementary material for: Letter contrast sensitivity validation
Source: Ophthalmic Physiol Opt. 2025 Aug 16;45(6):1317–25. doi: 10.1111/opo.13555 (PMC12357228; doi:10.1111/opo.13555)
Supplement: Supplementary file 1 — Data S1: [file OPO-45-1317-s001.zip › opo13555-sup-0003-FigureS2.pdf]

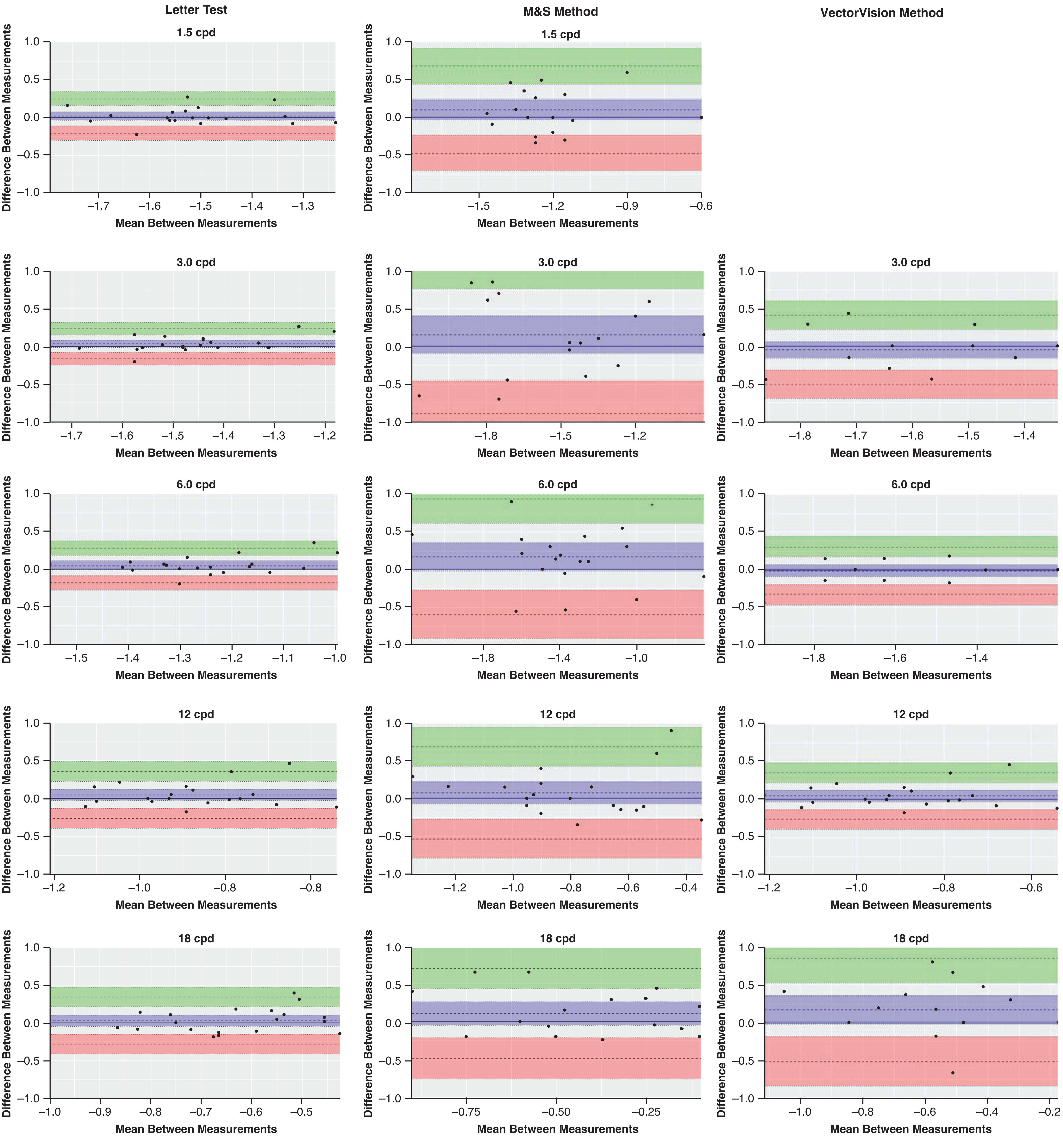

Supplemental Figure 3. Bland-Altman plots for mesopic conditions with glare. The dotted lines represent the upper and lower limit of the 95% confidence interval for bias (purple) and limits of agreement (green and red). cpd, cycles per degree of visual angle.
